# Comparing accuracy in voice-based assessments of biological speaker traits across speech types

**DOI:** 10.1038/s41598-023-49596-y

**Published:** 2023-12-27

**Authors:** Piotr Sorokowski, Agata Groyecka-Bernard, Tomasz Frackowiak, Aleksander Kobylarek, Piotr Kupczyk, Agnieszka Sorokowska, Michał Misiak, Anna Oleszkiewicz, Katarzyna Bugaj, Małgorzata Włodarczyk, Katarzyna Pisanski

**Affiliations:** 1 Institute of Psychology, University of Wrocław, Wrocław, Poland; 2 Institute of Pedagogy, University of Wrocław, Wrocław, Poland; 3 Being Human Lab, University of Wrocław, Wrocław, Poland; 4 Interdisciplinary Center Smell & Taste, Department of Otorhinolaryngology, Technische Universität Dresden, Dresden, Germany; 5 Laboratoire Dynamique du Langage, CNRS/Centre National de La Recherche Scientifique, Université Lyon 2, Lyon, France; 6 ENES Bioacoustics Research Lab, CRNL, University of Saint-Etienne, CNRS, Saint-Etienne, Inserm France

**Keywords:** Social evolution, Biological anthropology

## Abstract

Nonverbal acoustic parameters of the human voice provide cues to a vocaliser’s sex, age, and body size that are relevant in human social and sexual communication, and also increasingly so for computer-based voice recognition and synthesis technologies. While studies have shown some capacity in human listeners to gauge these biological traits from unseen speakers, it remains unknown whether speech complexity improves accuracy. Here, in over 200 vocalisers and 1500 listeners of both sexes, we test whether voice-based assessments of sex, age, height and weight vary from isolated vowels and words, to sequences of vowels and words, to full sentences or paragraphs. We show that while listeners judge sex and especially age more accurately as speech complexity increases, accuracy remains high across speech types, even for a single vowel sound. In contrast, the actual heights and weights of vocalisers explain comparatively less variance in listener’s assessments of body size, which do not vary systematically by speech type. Our results thus show that while more complex speech can improve listeners’ biological assessments, the gain is ecologically small, as listeners already show an impressive capacity to gauge speaker traits from extremely short bouts of standardised speech, likely owing to within-speaker stability in underlying nonverbal vocal parameters such as voice pitch. We discuss the methodological, technological, and social implications of these results.

## Introduction

Research on the biological and social communicative functions of nonverbal vocal parameters in the human voice has increased tremendously in the past two decades (for reviews see^1–3^). Hundreds of studies have measured the nonverbal properties of the human voice and/or examined how these affect listeners’ judgments in perception experiments. The overall aim is to understand what nonverbal features in the human voice can tell us about the speaker, from their emotional and motivational state or their physical traits such as their sex, age, and body size, to how such states and traits are perceived by others, as well as how vocal cues can offer insight into the communicative context of the vocal interaction^4,5^. The knowledge gained from such research has far reaching theoretical implications, including uncovering the evolutionary origins of acoustic communication and its adaptive social functions^6^. Indeed, vocal cues to sex, age and body size are important in the social and sexual communication systems of many animals^7^, including humans^8^, with ample evidence that selection has shaped the human voice to be highly sexually dimorphic and to signal, or exaggerate, body size^9,10^. Research into the communicative functions of the human voice also has many practical applications, for example improving voice recognition technologies (for review see^11^).

However, the extent to which human listeners’ voice-based judgments of biological or social traits reliably reflect the ‘true’ traits of vocalisers remains an open question, largely because findings vary across studies. It is not clear to what extent methodological differences play a role in this variation. Indeed, there remains surprisingly little consensus regarding the methods applied across voice studies, including those testing the exact same research question. Voice stimuli used in such research typically derive from audio recordings of vocalisers, but they vary widely in the type of speech recording content used for acoustic analysis and perception experiments, ranging from simple vowel sounds or nonverbal vocalisations to long trains of speech. This variation might affect the interpretation of results from past and future voice research, if, for example, longer and more complex speech stimuli elicit more accurate judgments of speakers on a given trait, or result in higher consensus among raters, compared to short stimuli such as vowels. On the other hand, if listeners’ voice-based judgments are not substantially affected by the type of speech produced by the vocaliser, this would suggest that studies using different kinds of speech may be comparable, and that any differences in their results are not likely due to the type of speech stimuli used. It would also offer further support that information encoded in the nonverbal vocal parameters of the human voice, such as in voice pitch, is relatively stable and largely impervious to changes in modal speech complexity^12,13^.

The present study may be the first to examine how different types of speech stimuli affect *accuracy* in listeners’ voice-based judgments of objective, biological speaker traits. However, previous work suggests that subjective speaker traits such as perceived attractiveness, dominance, or trustworthiness vary only slightly across speech types and thus that speakers are perceived in a similar way regardless of the complexity of their speech^13–15^.

Vowel sequences (e.g., “a i e o u”) have traditionally been a common type of speech stimulus used in studies of human nonverbal vocal communication, including perception studies examining listeners’ voice-based judgments of speakers^16–19^. Due to anatomical constraints on the vocal tract during vowel production^20^, sustained vowels provide the most standardised measures of formant frequencies, and thus are expected to most accurately reflect vocal tract length^7^ and body size^11,21^, which are biologically relevant traits in many animals, including humans^8^. The use of vowels can also be beneficial because it may mitigate the potential confounding effects of contextual and linguistic cues in the voice, for example arising from prosodic or sociolinguistic variation present in longer speech utterances^22^. Several basic vowel sounds are also shared across most languages (see the International Phonetic Alphabet) making them cross-culturally comparable. Moreover, as shown in a recent study by Pisanski and colleagues^12^, a person’s mean *f*_o_ (roughly perceived as their modal voice pitch) can also be reliably measured from simple vowel sounds. Thus, although they are very short in duration and largely lack prosodic contrasts, vowels constitute a standardised and comparable vocal stimulus, similar across languages and free of the potential confounds of verbal content.

In contrast, another commonly used type of speech stimulus is the Rainbow Passage^23^. Excerpts from this standardised and relatively long passage have been used in the speech sciences for several decades in part because it is phonetically balanced in American English, while also containing the vowel sounds present in most languages. Because of its length, most studies include only a portion of the passage^5,24,25^. Again, standardization makes this speech stimulus comparable across participants and studies of English speakers, however, the verbal content and the language of the passage make it less suitable for ecologically relevant investigations and cross-cultural comparisons. Indeed, the passage is almost always read, and the content is not socially relevant. Longer bouts of speech such as the Rainbow Passage may introduce greater variability in listeners’ judgments of vocaliser traits and states compared to vowel sounds, reducing inter-rater reliability. It is however also possible that listeners’ perceptions of a vocaliser based on a single vowel may change or reverse when presented with a longer bout of speech from the same speaker, owing either to additional information regarding prosodic and other nonverbal characteristics of the vocaliser’s voice, or owing to additional time to contemplate voice-based judgments. Numerous studies also rely on words or sentences as speech stimuli that may serve as a compromise between short vowels and long paragraphs. For instance, participants may be asked to count from 1 to 10 (e.g. ^26,27^,), to speak a short phrase (e.g. ^28,29^,), or to simply say a linguistic equivalent of “hello” (e.g. ^21,30^,).

While some types of vocal stimuli may indeed be more appropriate than others to test a specific hypothesis, integration of research findings becomes more complicated when a wide range of different stimuli are used to assess the exact same research question. For example, studies examining whether human listeners can reliably judge a person’s body size from their voice alone have most often used isolated vowels (e.g. ^17,19,31^, or single-syllable words (e.g. ^28,32,33^,), while others have used trains of speech (e.g. ^25,34^,), and more recently, even nonverbal vocalisations such as roars and screams^35,36^, to measure accuracy in listeners’ height judgments. Listeners’ apparent ability to reliably judge size from the human voice in fact varies considerably across such studies (^8,37^ for reviews), yet the degree to which the type of vocal stimulus contributes to this variability across studies is rarely taken into account.

Likewise, many studies that test analogous research questions regarding other biological, social or affective communicative functions of the human voice use different kinds of speech stimuli, and most often only use one type of speech per study (for reviews see^1,2,6,38^). In a recent paper, we showed that listener’s voice-based judgments of traits such as attractiveness, femininity and masculinity, trustworthiness, dominance and likeability can vary to some extent depending on the complexity of speech produced by the vocaliser, such that longer utterances explained the most shared variance in listeners’ judgments and elicited the highest ratings on all traits^13^. At the same time, listeners’ judgments showed a high degree of consistency across speech types within speakers, indicating that for these socially relevant traits, vocalisers were perceived largely similarly regardless of what they were saying^13^.

Testing the comparability of using different voice stimuli to answer the same research question is thus critical for the human voice sciences, particularly when mixed results arise^39–41^. It is crucial to assess not only the advantages of each method per se but also the advantages of each method *in comparison to other existing methods.* Here, we test the veracity in listeners’ judgments of four objective physical speaker traits – sex, age, height and weight – as a function of the type of speech produced by the same set of vocalisers, ranging from a single vowel sound to a full paragraph. By focusing on objective, biological traits with known values, we were able to test not only whether listeners’ judgments vary across speech types, but also whether listeners become more accurate in their judgments of biological speaker traits when provided with longer trains of speech.

## Methods

### Ethics statement

The study was conducted in accordance with the Declaration of Helsinki. Study protocols were accepted by the Ethics Committee at the Institute of Psychology, University of Wroclaw. All participants (vocalisers and listeners) provided informed consent prior to study inclusion. Vocalisers were informed that their voice recordings will be used for research purposes and that the recordings will be played to other participants.

### Participants

The sample of vocalisers consisted of 208 participants with an even sex ratio (49% women). The number of speakers (ca. 200) was determined as a trade-off between a sample that translates into a reasonable number of raters and one that allows us to detect inter-individual differences in biological traits of interest (namely age and body size) and nonverbal aspects of voice communication. In our large sample of speakers, vocaliser age ranged from 18 to 65 among men (M ± sd 30.4 ± 11.51 years) and 19 to 67 among women (35.39 ± 12.68 years). Height ranged from 161 to 197 cm among men (180.2 ± 6.14 cm) and 152 to 178 cm among women (165.65 ± 5.36 cm). Weight ranged from 58 to 130 kg among men (81.03 ± 13.24 kg) and 45 to 100 kg among women (65.62 ± 11.62 kg). Participants were recruited through snowball sampling and were not compensated for study participation.

A separate sex-balanced group of men and women participated as listeners in the rating experiment. Data were used from a final sample of 1561 listeners (aged M ± sd 31.60 ± 12.89 years, 47% women), after removing data from participants who failed entry or attention checks (see *Perception Experiments* below for exclusion details). All listeners reported normal hearing. The listeners were recruited via snowball sampling by researchers who posted recruitment ads on their social media profiles and around their city of residence, both inside and outside of the university. To increase the representativeness of our rater sample (i.e., including older individuals and non-students) we also recruited raters with the help of a research recruitment company. Raters were reimbursed in cash (for the cohort recruited via the recruitment firm) or through a lottery draw of small prizes such as pen drives (for all remaining participants).

The sample size of listeners was based on the sample size of vocalisers and voice stimuli. Achieving high inter-rater agreement in many voice-based judgements is possible with a sample size of approximately 15 listeners of each sex for each voice stimulus^42,43^. Thus, we ensured that each voice stimulus was judged by at least 15 listeners of each sex. Our large sample size of raters also ensured that we effectively captured inter-individual differences in listeners’ ratings, with the statistical power to test for possible effects of listener sex and age on this variability. Listeners were randomly assigned to assess only one of the following characteristics for each voice stimulus they heard: sex (*n* = 429 raters, M_age_ = 31.44, 47% women), age (*n* = 405 raters, M_age_ = 31.83, 46% women) height (*n* = 362 raters, M_age_ = 32.13, 48% women) or weight (*n* = 365 raters, M_age_ = 31.01, 46% women).

### Voice recording

Vocalisers took part in individual recording sessions. They were audio recorded in a quiet room using a Zoom H4n recorder positioned 10 cm from the mouth. They first familiarized themselves with a script containing a series of items (see *Voice stimuli*), and were then instructed to say each item aloud. Voice recordings were saved as WAV files at 96 kHz sampling frequency and 16-bit resolution, and later transferred to a laptop for acoustic analysis. Vocalisers completed a short survey self-reporting their sex and age. Their height and weight were measured using metric tape and a scale.

### Voice stimuli

Vocalisers each provided eight voice samples, among which six were used for the current study (English translation): (1) Vowels “a e i o u”: /a/ as in ‘bra’, /ɘ/ as in ‘bird’, /i/ as in ‘bee’, /ɔ/ as in ‘bot’, /u/ as in ‘boot’, with each vowel presented separately; (2) Vowels “a e i o u” presented simultaneously in a series; (3) One-syllable word “lat” (containing the vowel /a/ as in ‘bra’); (4) Counting from one to ten; (5) Single sentence greeting, “Good morning, I am from Poland”; and (6), the first six sentences of the Rainbow Passage^23^. Following the vast majority of voice studies in this domain (e.g. ^42,44^,), we chose to use only a one paragraph excerpt of the Rainbow Passage, rather than the full passage, to reduce the duration of playback experiments and subsequent fatigue in raters. While vowels were recorded only once as a sequence, this sequence was also parsed by vowel for the isolated-vowel condition. Two remaining passages involved free speech but were unrelated to the current study aims. Audio recordings were parsed into fragments containing one specific speech type each (i.e., 6 voice samples per vocaliser) for use in perception experiments. The relative length of the final speech items, computed as the number of syllables per item, was 1 syllable for each isolated vowel or word, 5 syllables for the series of vowels, 8 syllables for the greeting sentence, 16 syllables for counting, and 150 syllables for the Rainbow Passage.

### Perception experiment

Speech stimuli were presented to listeners via a custom-designed online application. Listeners completed the experiment at the university (individually or in small groups in a designated room with sufficient privacy to ensure independent responses), or online, with instructions to complete the study in a quiet space while focused only on this single task. Lab participants used Sennheiser HD 210 professional headphones. Participants who completed the playback online were instructed to use high quality headphones and to complete the study in a quiet environment without distractions. This was verified with hearing and attention tests. Before beginning, they were presented a test voice sample to ensure they can hear vocal stimuli and understand the study instructions. Eighty participants (< 5%) failed this entry test and their data were thus excluded from further analysis. Additionally, the experiment included one attention check during which listeners heard: “This is an attention check – please, mark 1”). Eleven participants (< 1%) were further excluded from analyses following an incorrect response.

For each listener, a sample of 10 vocalisers (5 per sex) was randomly drawn, with a set of 6 speech stimuli for each vocaliser, resulting in 60 judged voice stimuli per listener, played individually in a random order. Each speech stimulus was judged independently on a single trial and for a single physical characteristic. Listeners assessed sex on a two-alternative forced-choice scale (male/female), age in years (How old is this person?—ranging from 0 to 100) height in centimeters (How tall is this person?—ranging from 0 to 200) and weight in kilograms (How much does this person weigh?—ranging from 0 to 160).

### Statistical analysis

To test whether the type of voice stimulus affects the perceived properties of a vocaliser as well as the accuracy of listeners’ assessments, we first ran a series of Linear Mixed Models (LMMs). Data were nested within listeners and within vocalisers. All models were fitted with restricted maximum likelihood estimation (REML), and both listener and vocaliser identity were always included as random variables. In all models, continuous interaction components were group-mean centered (by listener) before being entered into the model. All other model parameters are described individually for each dimension in the Results. The estimates of fixed effects for all models as well as the estimates of covariance parameters can be found in Supplementary Materials. We present fully standardised estimates, wherein both predictors and outcome variables were standardised.

Due to strong sexual dimorphism in human voice pitch^19,45^ and a high degree of accurate sex recognition from the voice alone^1^, as also demonstrated by this study, sex can strongly bias other perceived vocaliser traits. All subsequent models were thus fitted separately for male and female vocaliser’s.

In LMMs, accuracy is shown by the effect of the actual vocaliser trait on the rated trait, whereas differences in accuracy across speech types are reflected by the actual trait * speech type interaction. Speech type was introduced as a categorical variable with six levels, split into five predictors, comparing each speech type to the longest speech type (the Rainbow Passage) as a reference point. To further investigate significant interactions, we ran regression analyses at the vocaliser level. We regressed actual age/height/weight on averaged assessments of these traits across all speech types. Finally, to further examine accuracy, we measured differences in the strengths of relationships between actual and assessed traits across all speech types using the web app http://quantpsy.org/corrtest/corrtest2.htm. First, each correlation coefficient was converted into a z-score using Fisher’s r-to-z transformation. Second, Steiger’s Equations^46^ were used to compute the asymptotic covariance of the estimates. These quantities are then used in an asymptotic z-test.

To calculate sex assessment, we ran a model with accuracy of sex assessment (1 – correct, 0 – incorrect) as a binary outcome variable. Speech type, vocaliser sex and age, and listener sex and age were included as fixed variables. Listener and vocaliser identity were treated as random variables.

### Transparency and openness

This article follows Journal Article Reporting Standards (JARS), including reports of how we determined our sample size, data exclusions, and all manipulations. All data, analysis code, and research materials are also freely and openly available at [https://osf.io/mcvf5/?view_only=b9c5bf95340345d8be5a04430dd6e75a]. Rater data were analyzed using Jamovi version 2.3.19.0. Graphs were created using RStudio v. 1.2.5042, ggplot package v. 3.3.3. This study’s design and its analysis were not pre-registered.

## Results

### Sex assessment

Speech type F(5, 24,860.79) = 56.25, *p* < 0.001, vocaliser sex F(1, 197.79) = 7.69, *p* = 0.006 and vocaliser age F(1, 203.43) = 4.91, *p* = 0.03 predicted sex assessments, with the sex of male and younger vocalisers most often correctly recognized (see Supplementary Table 1). No effects of listener sex or age were found (*p* = 0.27 and *p* = 0.83, respectively). As revealed by pairwise comparisons with Bonferroni correction for multiple comparisons, accuracy ratings were significantly lower for the shortest speech type (individual vowels) than for all other types. There were also small significant differences in accuracy of sex assessments comparing words to longer greetings or to the Rainbow Passage (see Supplementary Table 2 for standardised differences). Regardless of these statistically significant differences, accuracy in sex assessments was extremely high for all speech types, ranging from 95% for individual vowels and 98% for vowels presented simultaneously or single words, to 99% for counting, greetings and the Rainbow Passage (see Supplementary Table 3).

### Age assessment

In female vocalisers, speech type F(5, 11628.47)= 32.41, *p* <0.001, vocaliser age F(1, 107.46)= 199.57, *p* <0.001, and an interaction between these terms F(5, 11628.35)= 81.25, *p* <0.001 predicted age assessments, while listener sex and age did not (*p*=0.20 and 0.31, respectively). All standardised effects are presented in Supplementary Table 4. Pairwise differences among voice speech types with Bonferroni correction for multiple comparisons were significant in almost all pairs (see Supplementary Table 5). Nevertheless, the absolute difference between the speech type with the lowest (individual word, M=28.94) and highest (greeting, M=31.52) mean assessed age was small (see Supplementary Table 6). The fixed interaction effect of vocaliser's actual age and speech type was significant for individual vowels, vowels presented in a series, and single words (all vs. Rainbow Passage). This indicates that there were differences between the assessed and actual age of vocaliser’s depending on which speech type listeners judged, with Rainbow Passage eliciting the most accurate judgments (see Supplementary Table 4).

In male vocalisers, speech type F(5, 11482.62)= 8.49, *p* <0.001, vocaliser age F(1, 129.27)= 71.48, *p* <0.001, and listener age F(1, 382.77)= 13.69, *p* <0.001 predicted age assessments, with older listeners generally assessing vocalisers as older, and with no significant effect of listener sex (*p*=0.08). Here too, we found a significant interaction between speech type and vocaliser age among males F(5, 11482.40)= 50.28, *p* <0.001. All standardised effects are presented in Supplementary Table 6. Pairwise comparisons of age ratings were significant among almost all pairs (see Supplementary Table 7). However, as with female vocalisers, the absolute difference in age assessments between speech types with the lowest (counting, M=31.21) and highest (single word, M=32.51) assessed age was small (see Supplementary Table 8). Interaction effects revealed that age ratings based on the Rainbow Passage were again the most accurate compared to those based on all other speech types, except counting (see Supplementary Table 6).

**Figure 1:**
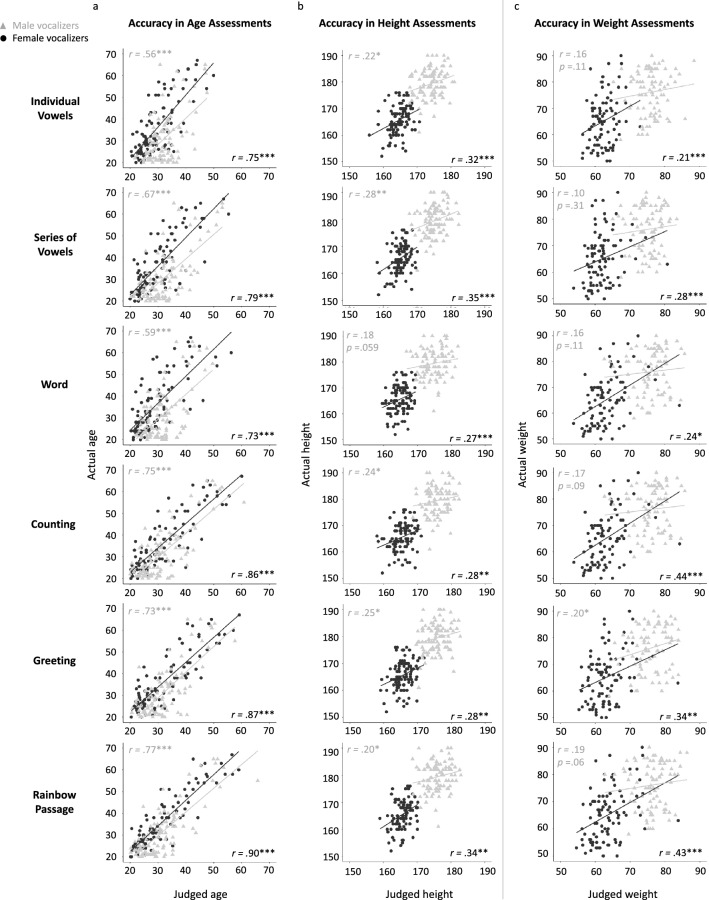
Accuracy in listener’s assessments of vocaliser age, height and weight. Vocalisers’ actual ages (years), heights (cm), and weights (kg) were regressed on listener’s assessments of their ages, heights, and weights, for each voice stimulus type. *Note*: Grey lines and triangular markers indicate male vocalisers, black lines and circular markers indicate female vocalisers. Pearson's *r* correlation coefficients and linear regression lines show the strength of each positive relationship. *p < 0.05, **p < 0.01, ***p < 0.001.

To assess how much variance in assessed age was explained by actual age, we correlated these values separately for each speech type. The relationship between actual and perceived age was moderate to strong^47^ for both male and female vocalisers, wherein actual age explained 31% to 81% of the variance in assessed age (Figure 1). However, stronger relationships were observed for the longest speech type, the Rainbow Passage (*r*= 0.90 for women and *r*= 0.77 for men), compared to individual vowels (*r*= 0.56 for men, *r*= 0.75 for women) and single words (*r*= 0.59 for men, *r*= 0.73 for women; see Figure 1). Readers who wish to assess the significance of differences between the individual correlation coefficients are directed to Table 19 located at the end of the supplementary materials. This table provides the relevant statistics for all variables described in the study.

### Height assessment

In female vocalisers, speech type F(5, 10383.88) = 9.29, *p*=0.001 and vocaliser actual height F(1, 107.02) = 21.78, *p*<0.001 predicted listeners’ height assessments, however the lack of an interaction effect F(5, 10383.88) = 0.36, *p* = 0.88 indicated that the amount of variance shared by assessed and actual height did not vary by speech type. Vocaliser age F(1, 98.66) = 1.07, *p* = 0.30, listener sex F(1, 358.77) = 1.57, *p* = 0.21 and listener age F(1, 359.35) = 1.35, *p*=0.24 also did not predict height assessments (see Supplementary Table 9). The differences between individual vowels and counting, greeting and Rainbow Passage, as well as between single words, greetings and Rainbow Passage were significant (see Supplementary Table 10). Nevertheless, the absolute difference between the highest and lowest mean estimated height (adjusted for covariates) was only 1.25 cm. The assessed mean height of female vocaliser’s ranged from 164.58 cm (individual vowels) to 165.83 (Rainbow Passage). See Supplementary Table 11 for the values for each speech type.

In male vocalisers, speech type F(5, 10,219.20) = 18.12, *p* < 0.001 and vocaliser actual height F(1, 112.29) = 22.08, *p* < 0.001 also predicted listeners’ height assessments. Like in females, the interaction effect was not significant F(5, 10,219.20) = 0.39, *p* = 0.86 nor were the effects of vocaliser age F(1, 105.23) = 0.40, *p* = 0.53 and listener sex F(1, 358.05) = 0.84, *p* = 0.36. A significant effect of listener age F(1, 358.59) = 17.15, *p* < 0.001 showed that younger listeners generally assessed male vocalisers as taller than did older listeners (see Supplementary Table 9). The differences between ratings were significant in almost all pairs of speech types (see Supplementary Table 10). The mean assessed height of male vocalisers ranged from 174.06 cm (individual vowels) to 176.04 cm (Rainbow Passage). The absolute difference between the highest and lowest mean estimated height (adjusted for covariates) was therefore less than 2 cm (see Supplementary Table 11).

Actual height explained a significant proportion of the variance in assessed height for each speech type, with the exception of single words produced by male vocalisers. Relationships were weak to moderate in strength, ranging from *r*=0.18 to *r*=0.28 for male vocalisers, and from *r*=0.27 to *r*=0.35 for females (see Figure 1). Thus, actual height could explain a maximum of 12% of the variance in assessed height. Both analysis of fixed effects in the main models and analysis of differences between correlations showed that the type of voice stimulus did not systematically predict accuracy in height assessments.

### Weight assessment

In female vocalisers, speech type F(5, 10488.878)= 14.80 *p* <0.001, the vocaliser’s actual weight F(1, 117.658)= 7.429, *p* =0.007, vocaliser age F(5, 98.958)= 75.210, *p* <0.001 and listener age F(5, 362.67)= 7.732, *p* =0.006, all predicted weight assessments. No effect of listener sex was found (*p*=0.68). We found an interaction between speech type and vocaliser actual weight, indicating differences in accuracy across speech types F(5, 10488.799)= 14.391, *p* <0.001. As presented in Supplementary Table 14, significant differences in accuracy were found between the longest speech stimulus (the Rainbow Passage) and all remaining speech types except for counting, with the Rainbow Passage eliciting more accurate ratings. In other words, in the case of the Rainbow Passage, actual weight shared more variance with assessed weight than in the case of other speech stimuli (except counting). The absolute difference between the speech type that elicited the lowest weight assessments (series of vowels) and the type that elicited the highest (Rainbow Passage) was significant (see Supplementary Table 15) but small, only 1.62 kg.

In male vocalisers, speech type F(5, 10295.83)= 7.361, *p* <0.001 predicted weight assessments, with no significant interaction effect F(5, 10295.61)= 0.645, *p* =0.665, and no effect of listener sex or age (*p*=0.08 in both cases). Actual weight did not significantly predict listeners’ assessments, however this relationship approached significance F(1, 111.245)= 3.648, *p* =0.059. Again, the absolute difference between speech types that elicited the lowest weight ratings (words) versus the highest (Rainbow Passage) was significant but low, at 1.47 kg.

Correlations between actual and assessed weight in male and female vocalisers were largely nonsignificant (except for greetings) and generally weak (ranging from *r*=0.1 to *r*=0.44). Correlations were also generally weaker for female (from *r*= 0.10 to 0.20) than male vocalisers (from *r*= 0.21 to 0.44, see Figure 1). Actual weight explained a maximum of 18% of variance in assessed weight. Both analysis of fixed effects in the main model and analysis of differences between correlations showed that the type of speech did not differentiate the accuracy of weight assessments in the case of male vocalisers. However, in female vocalisers, weight assessments from the Rainbow Passage (*r*= 0.43) and counting (r= 0.44) were more strongly predicted by the actual weights of vocalisers than were weight assessments based on single vowels (*r*= 0.21).

## Discussion

The present study was designed to test whether listeners’ voice-based judgments of a vocaliser’s sex, age, height and weight differ depending on what the speaker is saying, from single vowels to a paragraph, and whether accuracy in these judgments improves with longer and more complex speech stimuli. Based on a large sample of vocalisers and listeners, our results show only small differences across speech types in listeners’ assessments of these traits in the same group of vocalisers. While longer speech stimuli produce more accurate assessments of sex and especially of age in both sexes of vocalisers, and of weight in female vocalisers, these differences are small from an ecological perspective. Taken together our results show that while longer speech may provide listeners with a small amount of additional nonverbal cues and/or more time to judge speaker traits, listeners can manage to assess a great deal of information about a speaker’s biological traits from nothing more than a single vowel sound, as also observed for voice-based judgments of relatively more subjective speaker traits such as attractiveness and dominance^13^.

Listeners were extremely accurate in recognizing the sex of male and female vocalisers, achieving 99% accuracy from a full paragraph to 95% accuracy from a single vowel. Listeners were also accurate in recognizing the age of vocalisers. The actual age of vocalisers explained up to 81% of the variance in listeners’ assessments of age, with the most variance explained when judging a full paragraph (59–81%) and the least when judging single vowel sounds or words (31%–56%). Corroborating past work (^19^ for review), listeners were relatively less successful in reliably assessing the true body size of male and female vocalisers compared to assessments of sex or age. Indeed, the actual heights of vocalisers explained no more than 12%, and weight no more than 18%, of the variance in listener’s assessments within vocaliser sexes. These results align well with earlier acoustic analyses showing that key nonverbal vocal parameters (fundamental and formant frequencies) also explain only a small proportion of the variance in actual human heights and weights within sexes^19^, helping to explain the relatively low accuracy in listeners’ assessments of speaker body size from the voice. Interestingly, while listeners were not more accurate when judging height from longer than shorter stimuli, they tended to perceive vocalisers as generally taller (by 1.2 cm in female vocalisers and 2 cm in males) from longer bouts of speech. Listeners were moderately more accurate in judging the weights of female than of male vocalisers, and most accurate from longer bouts of speech such as a paragraph or counting (19%) than from individual vowels (9%). In the case of weight, like height, listeners perceived vocalisers as heavier from longer bouts of speech such as a paragraph (by 1.6 kg in female vocalisers and 1.5 kg in males).

The results show several general patterns that may be taken into account when researching human voice perception, including regarding methodology. Listeners were more likely to attribute lower values on all physical traits (sex, age, and body size) when presented with the least complex and shortest stimulus, individual vowels. Interestingly, our earlier work showed a similar pattern of results in listeners’ assessments of speaker attractiveness, health, dominance, trustworthiness and likeability – with ratings of these speaker traits generally increasing as the length of speech stimuli increased^13^. Importantly, by focusing here on objective speaker traits, we show that listeners were most accurate when judging sex, age, and to some extent weight from the longest and most complex stimuli (i.e. the Rainbow Passage) and least accurate when their judgments were based on simple sounds like vowels or words. On one hand these results suggest that, to increase the fidelity of human listeners’ judgments of physical traits, voice researchers may use longer speech bouts in their experimental designs. On the other hand, accuracy in listeners’ judgments, particularly of sex and age, was already high from simple vowel sounds and words. Hence there appears to be a trade-off between maximizing the length of vocal stimuli at the cost of prolonging experiments, with only a small gain in accuracy of judgments and response coherence across listeners. As our results indicate that the effect of stimulus length on accuracy is relatively linear, using stimuli of intermediate length and complexity (e.g., a greeting sentence) may offer a fair compromise to this trade-off.

Despite statistically significant differences observed across speech types, for example in the accuracy of judging sex, age, and women’s weight, it is difficult to gauge whether these small differences are ecologically relevant. The longest stimuli increased accuracy in sex judgments by only 4%, as sex judgments were already near ceiling, and increased the accuracy of weight judgments by only 10%, differences that may not be practically relevant in a real-world setting. Our research cannot provide an answer to this question. While even small differences may contribute to perceptual biases and discrimination in person perception, leading to serious societal consequences (e.g. ^48,49^,), in reality, voice-based judgments are typically based on more than a single word, and co-occur with visual and olfactory cues^50^. Vocal signals also often involve affective information, particularly in the production of emotional speech and nonverbal vocalisations such as laughs, cries, and moans that play an important role in human communication^51^ and were not included in our sample of voice stimuli. As such, the generalizability of these findings is limited to neutral speech. Moreover, it is important to note that listeners in our study judged the same speakers producing each of six neutral speech types, thus reducing noise in our data arising from inter-rater variance. While spillover effects cannot be entirely eliminated, speech stimuli were fully randomized, making it very unlikely that listeners could recognize individual speakers and recall ratings across a large number of trials.

This research offers novel evidence in support of the remarkable stability of speaker cues in the human voice, showing that vocal cues to a person’s sex, age, and body size are readily available to and utilized by human listeners, and largely impermeable to changes in speech structure and complexity. In future studies, researchers may test how perceptions of physical traits from a broader range of vocal stimuli influence listeners’ judgments and even their decisions and behaviours in real-world contexts. This may be especially relevant in the context of remote or virtual communication during which people may base their judgments of others more heavily on vocal cues, for instance, during video conferencing, online hiring, remote classes or gaming. The extent to which the results of this research generalize beyond human listeners to computer-based detection of biological markers from human speech is, in our opinion, a critical avenue for further investigation.

### Supplementary Information

Supplementary Information.

## Data Availability

The datasets generated and analysed during the current study are available in the Open Science Framework repository, https://osf.io/mcvf5/?view_only=b9c5bf95340345d8be5a04430dd6e75a.
