# Supplementary material for: Comparing accuracy in voice-based assessments of biological speaker traits across speech types
Source: Sci Rep. 2023 Dec 27;13:22989. doi: 10.1038/s41598-023-49596-y (PMC10752881; doi:10.1038/s41598-023-49596-y)
Supplement: Supplementary file 1 — Supplementary Information. [file 41598_2023_49596_MOESM1_ESM.docx]

Supplementary Materials: *Comparing accuracy in voice-based assessments of biological speaker traits across speech types*

The following coding is valid through the document:

1 – single vowels

2 – vowels

3 – single word

4 – counting

5 – greeting

6 – Rainbow Passage

Table 1. Estimates of fixed and random effects in a model predicting accuracy in sex ratings by type of speech and covariates.

|  |  | |  |  | |  |  |  |  |
| --- | --- | --- | --- | --- | --- | --- | --- | --- | --- |
| Fixed effects | |  | | |  |  | 95% CI | |  |
|  | |  | | | Estimate | SE | Lower | Upper | p |
| Intercept | |  | | | -0.001 | 0.018 | -0.036 | 0.034 | 0.944 |
| Speech type | | 1 vs. 6 | | | -0.277 | 0.020 | -0.317 | -0.237 | < .001 |
|  | | 2 vs. 6 | | | -0.035 | 0.020 | -0.075 | 0.005 | 0.085 |
|  | | 3 vs. 6 | | | -0.081 | 0.020 | -0.121 | -0.041 | < .001 |
|  | | 4 vs. 6 | | | -0.005 | 0.020 | -0.045 | 0.035 | 0.808 |
|  | | 5 vs. 6 | | | 0.005 | 0.020 | -0.035 | 0.045 | 0.803 |
| Vocaliser’s sex | |  | | | 0.033 | 0.012 | 0.010 | 0.057 | 0.006 |
| Vocaliser’s age | |  | | | -0.027 | 0.012 | -0.051 | -0.003 | 0.028 |
| Listener’s sex | |  | | | -0.017 | 0.015 | -0.047 | 0.013 | 0.266 |
| Listener’s age | |  | | | 0.003 | 0.015 | -0.026 | 0.033 | 0.826 |
| Random effects | |  | | | Variance |  | ICC |  |  |
| Listener’s ID | |  | | | 0.161 |  | 0.255 |  |  |
| Vocalizer’s ID | |  | | | 0.100 |  | 0.175 |  |  |
| Residuals | |  | | | 0.471 |  |  |  |  |

| Table 2. Standardized differences between accuracy in sex recognition across speech types. | | | | | | | | | | | | | |
| --- | --- | --- | --- | --- | --- | --- | --- | --- | --- | --- | --- | --- | --- |
| **Comparison** | | | | | |  | | | | | | | |
| **Speech type** | |  | | **Speech type** | | **Difference** | | **SE** | | **z** | | **p_bonferroni_** | |
| single vowels |  | - |  | vowels |  | -0.242 |  | 0.020 |  | -11.823 |  | < .001 |  |
| single vowels |  | - |  | single word |  | -0.197 |  | 0.020 |  | -9.605 |  | < .001 |  |
| single vowels |  | - |  | counting |  | -0.272 |  | 0.020 |  | -13.302 |  | < .001 |  |
| single vowels |  | - |  | greeting |  | -0.282 |  | 0.020 |  | -13.793 |  | < .001 |  |
| single vowels |  | - |  | Rainbow Passage |  | -0.277 |  | 0.020 |  | -13.544 |  | < .001 |  |
| vowels |  | - |  | single word |  | 0.045 |  | 0.020 |  | 2.218 |  | 0.399 |  |
| vowels |  | - |  | counting |  | -0.030 |  | 0.020 |  | -1.479 |  | 1.000 |  |
| vowels |  | - |  | greeting |  | -0.040 |  | 0.020 |  | -1.971 |  | 0.731 |  |
| vowels |  | - |  | Rainbow Passage |  | -0.035 |  | 0.020 |  | -1.722 |  | 1.000 |  |
| single word |  | - |  | counting |  | -0.076 |  | 0.020 |  | -3.697 |  | 0.003 |  |
| single word |  | - |  | greeting |  | -0.086 |  | 0.020 |  | -4.188 |  | < .001 |  |
| single word |  | - |  | Rainbow Passage |  | -0.081 |  | 0.020 |  | -3.939 |  | 0.001 |  |
| counting |  | - |  | greeting |  | -0.010 |  | 0.020 |  | -0.491 |  | 1.000 |  |
| counting |  | - |  | Rainbow Passage |  | -0.005 |  | 0.020 |  | -0.243 |  | 1.000 |  |
| greeting |  | - |  | Rainbow Passage |  | 0.005 |  | 0.020 |  | 0.249 |  | 1.000 |  |
| Table 3. Estimated marginal means of accuracy in sex recognition across speech types.   \| **Speech type** \| **Mean** \| **SE** \| **Lower** \| **Upper** \| \| --- \| --- \| --- \| --- \| --- \| \| single vowels \| 0.950 \| 0.003 \| 0.944 \| 0.956 \| \| vowels \| 0.984 \| 0.003 \| 0.978 \| 0.990 \| \| single word \| 0.978 \| 0.003 \| 0.972 \| 0.984 \| \| counting \| 0.988 \| 0.003 \| 0.982 \| 0.995 \| \| greeting \| 0.990 \| 0.003 \| 0.984 \| 0.996 \| \| Rainbow Passage \| 0.989 \| 0.003 \| 0.983 \| 0.995 \| | | | | | | | | | | | | | |

|  |  |  |  |  | |  |  |  |  | |  |
| --- | --- | --- | --- | --- | --- | --- | --- | --- | --- | --- | --- |
| Table 4. Estimates of fixed and random effects in a model predicting age ratings by type of speech, Vocaliser’s age (centered), listener’s sex, listener’s age and type of speech * Vocaliser’s age (centered) interaction. | | | | | | | | | | | |
| Fixed effects |  |  |  | 95% CI | |  |  |  | 95% CI | |  |
|  |  | Estimate | SE | Lower | Upper | p | Estimate | SE | Lower | Upper | p |
|  |  | Female vocalizers | | | | | Male vocalizers | | | | |
| Intercept |  | -0.002 | 0.038 | -0.076 | 0.072 | 0.957 | 0.001 | 0.041 | -0.080 | 0.082 | 0.990 |
| Speech type | 1 vs. 6 | -0.124 | 0.022 | -0.166 | -0.081 | < .001 | 0.097 | 0.023 | 0.051 | 0.143 | < .001 |
|  | 2 vs. 6 | -0.115 | 0.022 | -0.157 | -0.072 | < .001 | 0.110 | 0.023 | 0.064 | 0.155 | < .001 |
|  | 3 vs. 6 | -0.175 | 0.022 | -0.217 | -0.133 | < .001 | 0.025 | 0.023 | -0.020 | 0.071 | 0.275 |
|  | 4 vs. 6 | 0.012 | 0.022 | -0.031 | 0.054 | 0.588 | -0.001 | 0.023 | -0.046 | 0.045 | 0.982 |
|  | 5 vs. 6 | 0.033 | 0.022 | -0.009 | 0.076 | 0.121 | 0.056 | 0.023 | 0.011 | 0.102 | 0.015 |
| Vocaliser’s age |  | 0.402 | 0.028 | 0.347 | 0.458 | < .001 | 0.260 | 0.031 | 0.200 | 0.321 | < .001 |
| Vocalisers age*speech type | *1 vs. 6 | -0.319 | 0.022 | -0.361 | -0.276 | < .001 | -0.288 | 0.023 | -0.333 | -0.242 | < .001 |
|  | *2 vs. 6 | -0.215 | 0.022 | -0.257 | -0.172 | < .001 | -0.152 | 0.023 | -0.198 | -0.107 | < .001 |
|  | *3 vs. 6 | -0.263 | 0.022 | -0.305 | -0.221 | < .001 | -0.252 | 0.023 | -0.298 | -0.206 | < .001 |
|  | *4 vs. 6 | -0.039 | 0.022 | -0.081 | 0.003 | 0.071 | -0.029 | 0.023 | -0.075 | 0.016 | 0.204 |
|  | *5 vs. 6 | -0.029 | 0.022 | -0.071 | 0.013 | 0.176 | -0.100 | 0.023 | -0.145 | -0.054 | < .001 |
| Listener’s sex |  | -0.027 | 0.021 | -0.069 | 0.014 | 0.201 | -0.041 | 0.024 | -0.087 | 0.005 | 0.084 |
| Listener’s age |  | 0.021 | 0.021 | -0.020 | 0.063 | 0.313 | 0.087 | 0.024 | 0.041 | 0.134 | < .001 |
| Random effects |  | Variance |  | ICC |  |  | Estimate | SE | p |  |  |
| Listener’s ID |  | 0.161 |  | 0.255 |  |  | 0.201 |  | 0.271 |  |  |
| Vocalizer’s ID |  | 0.100 |  | 0.175 |  |  | 0.123 |  | 0.186 |  |  |
| Residuals |  | 0.471 |  |  |  |  | 0.540 |  |  |  |  |

| Table 5. Standardized differences between perceived age of the vocalizers across speech types in females. | | | | | | | | | | | | | | | |
| --- | --- | --- | --- | --- | --- | --- | --- | --- | --- | --- | --- | --- | --- | --- | --- |
| **Comparison** | | | | | |  | | | | | | | | | |
| **Speech type** | |  | | **Speech type** | | **Difference** | | **SE** | | **t** | | **df** | | **p_bonferroni_** | |
| single vowels |  | - |  | Vowels |  | -0.009 |  | 0.022 |  | -0.407 |  | 11628.718 |  | 1.000 |  |
| single vowels |  | - |  | single word |  | 0.051 |  | 0.022 |  | 2.386 |  | 11628.961 |  | 0.256 |  |
| single vowels |  | - |  | Counting |  | -0.135 |  | 0.022 |  | -6.268 |  | 11628.712 |  | < .001 |  |
| single vowels |  | - |  | Greeting |  | -0.157 |  | 0.022 |  | -7.279 |  | 11628.388 |  | < .001 |  |
| single vowels |  | - |  | Rainbow Passage |  | -0.124 |  | 0.022 |  | -5.727 |  | 11628.717 |  | < .001 |  |
| vowels |  | - |  | single word |  | 0.060 |  | 0.022 |  | 2.792 |  | 11628.318 |  | 0.079 |  |
| vowels |  | - |  | Counting |  | -0.126 |  | 0.022 |  | -5.859 |  | 11628.293 |  | < .001 |  |
| vowels |  | - |  | Greeting |  | -0.148 |  | 0.022 |  | -6.869 |  | 11628.387 |  | < .001 |  |
| vowels |  | - |  | Rainbow Passage |  | -0.115 |  | 0.022 |  | -5.318 |  | 11628.283 |  | < .001 |  |
| single word |  | - |  | Counting |  | -0.187 |  | 0.022 |  | -8.650 |  | 11628.305 |  | < .001 |  |
| single word |  | - |  | Greeting |  | -0.208 |  | 0.022 |  | -9.660 |  | 11628.530 |  | < .001 |  |
| single word |  | - |  | Rainbow Passage |  | -0.175 |  | 0.022 |  | -8.109 |  | 11628.313 |  | < .001 |  |
| counting |  | - |  | Greeting |  | -0.022 |  | 0.022 |  | -1.009 |  | 11628.390 |  | 1.000 |  |
| counting |  | - |  | Rainbow Passage |  | 0.012 |  | 0.022 |  | 0.541 |  | 11628.291 |  | 1.000 |  |
| greeting |  | - |  | Rainbow Passage |  | 0.033 |  | 0.022 |  | 1.550 |  | 11628.391 |  | 1.000 |  |
|  | | | | | | | | | | | | | | | |

|  |  |  |  |  |  |  |
| --- | --- | --- | --- | --- | --- | --- |
|  |  |  |  |  |  |  |

| Table 6. Estimated marginal means of perceived age of the vocalizers depending on speech type in females. | | | | | | | | | | | |
| --- | --- | --- | --- | --- | --- | --- | --- | --- | --- | --- | --- |
|  | | | | | | | | **95% Confidence Interval** | | | |
| **Speech type** | | **Mean** | | **SE** | | **df** | | **Lower** | | **Upper** | |
| single vowels |  | 29.580 |  | 0.499 |  | 185.879 |  | 28.595 |  | 30.565 |  |
| vowels |  | 29.688 |  | 0.499 |  | 185.989 |  | 28.703 |  | 30.673 |  |
| single word |  | 28.942 |  | 0.499 |  | 186.019 |  | 27.957 |  | 29.927 |  |
| counting |  | 31.251 |  | 0.499 |  | 185.988 |  | 30.266 |  | 32.237 |  |
| greeting |  | 31.520 |  | 0.499 |  | 185.931 |  | 30.535 |  | 32.505 |  |
| Rainbow Passage |  | 31.108 |  | 0.499 |  | 185.986 |  | 30.123 |  | 32.093 |  |
|  | | | | | | | | | | | |

| Table 7. Standardized differences between perceived age of the vocalizers across speech types in males. | | | | | | | | | | | | | | | |
| --- | --- | --- | --- | --- | --- | --- | --- | --- | --- | --- | --- | --- | --- | --- | --- |
| **Comparison** | | | | | |  | | | | | | | | | |
| **Speech type** | |  | | **Speech type** | | **Difference** | | **SE** | | **t** | | **df** | | **p_bonferroni_** | |
| single vowels |  | - |  | Vowels |  | -0.013 |  | 0.023 |  | -0.550 |  | 11482.691 |  | 1.000 |  |
| single vowels |  | - |  | single word |  | 0.072 |  | 0.023 |  | 3.081 |  | 11482.693 |  | 0.031 |  |
| single vowels |  | - |  | Counting |  | 0.098 |  | 0.023 |  | 4.196 |  | 11483.266 |  | < .001 |  |
| single vowels |  | - |  | Greeting |  | 0.041 |  | 0.023 |  | 1.752 |  | 11483.266 |  | 1.000 |  |
| single vowels |  | - |  | Rainbow Passage |  | 0.097 |  | 0.023 |  | 4.172 |  | 11482.691 |  | < .001 |  |
| vowels |  | - |  | single word |  | 0.084 |  | 0.023 |  | 3.632 |  | 11482.381 |  | 0.004 |  |
| vowels |  | - |  | Counting |  | 0.110 |  | 0.023 |  | 4.749 |  | 11482.528 |  | < .001 |  |
| vowels |  | - |  | Greeting |  | 0.054 |  | 0.023 |  | 2.303 |  | 11482.528 |  | 0.320 |  |
| vowels |  | - |  | Rainbow Passage |  | 0.110 |  | 0.023 |  | 4.724 |  | 11482.372 |  | < .001 |  |
| single word |  | - |  | Counting |  | 0.026 |  | 0.023 |  | 1.116 |  | 11482.527 |  | 1.000 |  |
| single word |  | - |  | Greeting |  | -0.031 |  | 0.023 |  | -1.330 |  | 11482.527 |  | 1.000 |  |
| single word |  | - |  | Rainbow Passage |  | 0.025 |  | 0.023 |  | 1.092 |  | 11482.381 |  | 1.000 |  |
| counting |  | - |  | Greeting |  | -0.057 |  | 0.023 |  | -2.446 |  | 11482.372 |  | 0.217 |  |
| counting |  | - |  | Rainbow Passage |  | -0.001 |  | 0.023 |  | -0.023 |  | 11482.528 |  | 1.000 |  |
| greeting |  | - |  | Rainbow Passage |  | 0.056 |  | 0.023 |  | 2.423 |  | 11482.528 |  | 0.231 |  |
|  | | | | | | | | | | | | | | | |

| Table 8. Estimated marginal means of perceived age of the vocalizers depending on speech type in males. | | | | | | | | | | | |
| --- | --- | --- | --- | --- | --- | --- | --- | --- | --- | --- | --- |
|  | | | | | | | | **95% Confidence Interval** | | | |
| **Speech type** | | **Mean** | | **SE** | | **df** | | **Lower** | | **Upper** | |
| single vowels |  | 32.362 |  | 0.519 |  | 214.402 |  | 31.339 |  | 33.384 |  |
| vowels |  | 32.513 |  | 0.519 |  | 214.286 |  | 31.491 |  | 33.535 |  |
| single word |  | 31.519 |  | 0.519 |  | 214.284 |  | 30.497 |  | 32.542 |  |
| counting |  | 31.214 |  | 0.519 |  | 214.220 |  | 30.192 |  | 32.237 |  |
| greeting |  | 31.883 |  | 0.519 |  | 214.220 |  | 30.861 |  | 32.905 |  |
| Rainbow Passage |  | 31.220 |  | 0.519 |  | 214.286 |  | 30.198 |  | 32.242 |  |
| Note. Estimated means are estimated keeping constant other effects in the model to the mean | | | | | | | | | | | |
|  | | | | | | | | | | | |

| Table 9. Estimates of fixed and random effects in a model predicting height ratings by type of speech, Vocaliser’s height (centered), Vocaliser’s age, listener’s sex, listener’s age and type of speech * Vocaliser’s height (centered) interaction. | | | | | | | | | | | |
| --- | --- | --- | --- | --- | --- | --- | --- | --- | --- | --- | --- |
| Fixed effects |  |  |  | 95% CI | |  |  | | 95% CI | |  |
|  |  | Estimate | SE | Lower | Upper | p | Estimate | SE | Lower | Upper | p |
| Female vocalizers | | | | | | | Male vocalizers | | | | |
| Intercept |  | 0.006 | 0.036 | -0.064 | -0.064 | 0.863 | 0.003 | 0.039 | -0.073 | 0.080 | 0.930 |
| Speech type | 1 vs. 6 | -0.150 | 0.026 | -0.201 | -0.201 | < .001 | -0.199 | 0.024 | -0.246 | -0.151 | < .001 |
|  | 2 vs. 6 | -0.075 | 0.026 | -0.127 | -0.127 | 0.004 | -0.095 | 0.024 | -0.143 | -0.048 | < .001 |
|  | 3 vs. 6 | -0.108 | 0.026 | -0.159 | -0.159 | < .001 | -0.120 | 0.024 | -0.167 | -0.072 | < .001 |
|  | 4 vs. 6 | -0.046 | 0.026 | -0.097 | -0.097 | 0.080 | -0.026 | 0.024 | -0.074 | 0.021 | 0.279 |
|  | 5 vs. 6 | -0.019 | 0.026 | -0.070 | -0.070 | 0.478 | -0.044 | 0.024 | -0.092 | 0.003 | 0.068 |
| Vocaliser’s height |  | 0.080 | 0.017 | 0.046 | 0.046 | < .001 | 0.083 | 0.018 | 0.048 | 0.117 | < .001 |
| Vocalisers height*speech type | *1 vs. 6 | -0.001 | 0.026 | -0.052 | 0.051 | 0.981 | 0.007 | 0.024 | -0.040 | 0.055 | 0.768 |
|  | *2 vs. 6 | 0.005 | 0.026 | -0.046 | 0.056 | 0.857 | 0.016 | 0.024 | -0.032 | 0.063 | 0.513 |
|  | *3 vs. 6 | -0.026 | 0.026 | -0.077 | 0.025 | 0.320 | -0.015 | 0.024 | -0.062 | 0.033 | 0.546 |
|  | *4 vs. 6 | -0.005 | 0.026 | -0.056 | 0.046 | 0.856 | 0.007 | 0.024 | -0.040 | 0.055 | 0.758 |
|  | *5 vs. 6 | -0.012 | 0.026 | -0.063 | 0.039 | 0.649 | 0.011 | 0.024 | -0.037 | 0.058 | 0.657 |
| Vocaliser’s age |  | -0.001 | 0.001 | -0.004 | 0.001 | 0.304 | -0.001 | 0.002 | -0.004 | 0.002 | 0.527 |
| Listener’s sex |  | 0.041 | 0.033 | -0.023 | 0.105 | 0.210 | 0.032 | 0.035 | -0.037 | 0.102 | 0.361 |
| Listener’s age |  | -0.038 | 0.033 | -0.102 | 0.026 | 0.247 | -0.146 | 0.035 | -0.215 | -0.077 | < .001 |
| Random effects |  | Variance |  | ICC |  |  | Variance |  | ICC |  |  |
| Listener’s ID |  | 0.359 |  | 0.368 |  |  | 0.428 |  | 0.451 |  |  |
| Vocalizer’s ID |  | 0.023 |  | 0.036 |  |  | 0.032 |  | 0.058 |  |  |
| Residuals |  | 0.617 |  |  |  |  | 0.522 |  |  |  |  |

| Table 10. Standardized differences between perceived height of the vocalizers across speech types in females. | | | | | | | | | | | | | | | | | | |  |
| --- | --- | --- | --- | --- | --- | --- | --- | --- | --- | --- | --- | --- | --- | --- | --- | --- | --- | --- | --- |
| **Comparison** | | | | | |  | | | | | | | | | | | | |  |
| **Speech type** | |  | | **Speech type** | | **Difference** | | **SE** | | | **t** | | **df** | | | **p_bonferroni_** | | |  |
| single vowels |  | - |  | vowels |  | -0.074 |  | | 0.026 |  | | -2.839 |  | 10383.879 |  | | 0.068 |  | |
| single vowels |  | - |  | single word |  | -0.042 |  | | 0.026 |  | | -1.610 |  | 10383.879 |  | | 1.000 |  | |
| single vowels |  | - |  | counting |  | -0.104 |  | | 0.026 |  | | -3.979 |  | 10383.879 |  | | 0.001 |  | |
| single vowels |  | - |  | greeting |  | -0.131 |  | | 0.026 |  | | -5.020 |  | 10383.879 |  | | < .001 |  | |
| single vowels |  | - |  | Rainbow Passage |  | -0.150 |  | | 0.026 |  | | -5.729 |  | 10383.879 |  | | < .001 |  | |
| vowels |  | - |  | single word |  | 0.032 |  | | 0.026 |  | | 1.230 |  | 10383.879 |  | | 1.000 |  | |
| vowels |  | - |  | counting |  | -0.030 |  | | 0.026 |  | | -1.140 |  | 10383.879 |  | | 1.000 |  | |
| vowels |  | - |  | greeting |  | -0.057 |  | | 0.026 |  | | -2.181 |  | 10383.879 |  | | 0.438 |  | |
| vowels |  | - |  | Rainbow Passage |  | -0.075 |  | | 0.026 |  | | -2.890 |  | 10383.879 |  | | 0.058 |  | |
| single word |  | - |  | counting |  | -0.062 |  | | 0.026 |  | | -2.370 |  | 10383.879 |  | | 0.267 |  | |
| single word |  | - |  | greeting |  | -0.089 |  | | 0.026 |  | | -3.410 |  | 10383.879 |  | | 0.010 |  | |
| single word |  | - |  | Rainbow Passage |  | -0.108 |  | | 0.026 |  | | -4.120 |  | 10383.879 |  | | < .001 |  | |
| counting |  | - |  | greeting |  | -0.027 |  | | 0.026 |  | | -1.041 |  | 10383.879 |  | | 1.000 |  | |
| counting |  | - |  | Rainbow Passage |  | -0.046 |  | | 0.026 |  | | -1.750 |  | 10383.879 |  | | 1.000 |  | |
| greeting |  | - |  | Rainbow Passage |  | -0.019 |  | | 0.026 |  | | -0.709 |  | 10383.879 |  | | 1.000 |  | |
|  | | | | | | | | | | | | | | | | | | |  |

| Table 11. Estimated marginal means of perceived height of the vocalizers depending on speech type in females | | | | | | | | | | | |
| --- | --- | --- | --- | --- | --- | --- | --- | --- | --- | --- | --- |
|  | | | | | | | | **95% Confidence Interval** | | | |
| **Speech type** | | **Mean** | | **SE** | | **df** | | **Lower** | | **Upper** | |
| single vowels |  | 164.584 |  | 0.329 |  | 601.534 |  | 163.939 |  | 165.230 |  |
| vowels |  | 165.199 |  | 0.329 |  | 601.534 |  | 164.553 |  | 165.844 |  |
| single word |  | 164.933 |  | 0.329 |  | 601.534 |  | 164.287 |  | 165.578 |  |
| counting |  | 165.446 |  | 0.329 |  | 601.534 |  | 164.800 |  | 166.091 |  |
| greeting |  | 165.671 |  | 0.329 |  | 601.534 |  | 165.026 |  | 166.317 |  |
| Rainbow Passage |  | 165.825 |  | 0.329 |  | 601.534 |  | 165.179 |  | 166.470 |  |
| Note. Estimated means are estimated averaging across interacting variables | | | | | | | | | | | |
|  | | | | | | | | | | | |

| Table 12. Standardized differences between perceived height of the vocalizers across speech types in males. | | | | | | | | | | | | | | | |
| --- | --- | --- | --- | --- | --- | --- | --- | --- | --- | --- | --- | --- | --- | --- | --- |
| **Comparison** | | | | | |  | | | | | | | | | |
| **Speech type** | |  | | **Speech type** | | **Difference** | | **SE** | | **t** | | **df** | | **p_bonferroni_** | |
| single vowels |  | - |  | vowels |  | -0.103 |  | 0.024 |  | -4.274 |  | 10219.198 |  | < .001 |  |
| single vowels |  | - |  | single word |  | -0.079 |  | 0.024 |  | -3.273 |  | 10219.198 |  | 0.016 |  |
| single vowels |  | - |  | counting |  | -0.173 |  | 0.024 |  | -7.137 |  | 10219.198 |  | < .001 |  |
| single vowels |  | - |  | greeting |  | -0.155 |  | 0.024 |  | -6.393 |  | 10219.198 |  | < .001 |  |
| single vowels |  | - |  | Rainbow Passage |  | -0.199 |  | 0.024 |  | -8.219 |  | 10219.214 |  | < .001 |  |
| vowels |  | - |  | single word |  | 0.024 |  | 0.024 |  | 1.000 |  | 10219.198 |  | 1.000 |  |
| vowels |  | - |  | counting |  | -0.069 |  | 0.024 |  | -2.863 |  | 10219.198 |  | 0.063 |  |
| vowels |  | - |  | greeting |  | -0.051 |  | 0.024 |  | -2.119 |  | 10219.198 |  | 0.511 |  |
| vowels |  | - |  | Rainbow Passage |  | -0.095 |  | 0.024 |  | -3.946 |  | 10219.214 |  | 0.001 |  |
| single word |  | - |  | counting |  | -0.093 |  | 0.024 |  | -3.863 |  | 10219.198 |  | 0.002 |  |
| single word |  | - |  | greeting |  | -0.075 |  | 0.024 |  | -3.120 |  | 10219.198 |  | 0.027 |  |
| single word |  | - |  | Rainbow Passage |  | -0.120 |  | 0.024 |  | -4.946 |  | 10219.214 |  | < .001 |  |
| counting |  | - |  | greeting |  | 0.018 |  | 0.024 |  | 0.744 |  | 10219.198 |  | 1.000 |  |
| counting |  | - |  | Rainbow Passage |  | -0.026 |  | 0.024 |  | -1.084 |  | 10219.214 |  | 1.000 |  |
| greeting |  | - |  | Rainbow Passage |  | -0.044 |  | 0.024 |  | -1.827 |  | 10219.214 |  | 1.000 |  |
|  | | | | | | | | | | | | | | | |

| Table 13. Estimated marginal means of perceived height of the vocalizers depending on speech type in males. | | | | | | | | | | | |
| --- | --- | --- | --- | --- | --- | --- | --- | --- | --- | --- | --- |
|  | | | | | | | | **95% Confidence Interval** | | | |
| **Speech type** | | **Mean** | | **SE** | | **df** | | **Lower** | | **Upper** | |
| single vowels |  | 174.062 |  | 0.420 |  | 569.702 |  | 173.237 |  | 174.887 |  |
| vowels |  | 175.090 |  | 0.420 |  | 569.702 |  | 174.265 |  | 175.915 |  |
| single word |  | 174.849 |  | 0.420 |  | 569.702 |  | 174.025 |  | 175.674 |  |
| counting |  | 175.779 |  | 0.420 |  | 569.702 |  | 174.954 |  | 176.603 |  |
| greeting |  | 175.600 |  | 0.420 |  | 569.702 |  | 174.775 |  | 176.424 |  |
| Rainbow Passage |  | 176.039 |  | 0.420 |  | 569.807 |  | 175.215 |  | 176.864 |  |
|  | | | | | | | | | | | |

Table 14. Estimates of fixed and random effects in a model predicting weight ratings by type of speech, Vocaliser’s weight (centered), Vocaliser’s age, listener’s sex, listener’s age and type of speech * Vocaliser’s weight (centered) interaction.

| Female vocalizers | | | | | | | Male vocalizers | | | | | | | | | | |
| --- | --- | --- | --- | --- | --- | --- | --- | --- | --- | --- | --- | --- | --- | --- | --- | --- | --- |
| Fixed effects | |  |  |  | 95% CI | | | |  |  |  |  | | 95% CI | |  |  |
|  | |  | Estimate | SE | Lower | Upper | p | Estimate | | | | SE | | Lower | Upper | p | |
| Intercept | |  | 0.006 | 0.037 | -0.067 | 0.079 | 0.866 | -0.008 | | | | 0.041 | | -0.088 | 0.072 | 0.843 | |
| Speech type | | 1 vs. 6 | -0.147 | 0.025 | -0.197 | -0.097 | < .001 | -0.054 | | | | 0.025 | | -0.102 | -0.005 | 0.030 | |
|  | | 2 vs. 6 | -0.167 | 0.026 | -0.217 | -0.117 | < .001 | -0.009 | | | | 0.025 | | -0.058 | 0.039 | 0.701 | |
|  | | 3 vs. 6 | -0.175 | 0.026 | -0.225 | -0.125 | < .001 | -0.131 | | | | 0.025 | | -0.179 | -0.082 | < .001 | |
|  | | 4 vs. 6 | -0.070 | 0.026 | -0.120 | -0.020 | 0.006 | -0.022 | | | | 0.025 | | -0.070 | 0.027 | 0.379 | |
|  | | 5 vs. 6 | -0.068 | 0.026 | -0.118 | -0.018 | 0.008 | -0.046 | | | | 0.025 | | -0.094 | 0.003 | 0.063 | |
| Vocaliser’s weight | |  | 0.074 | 0.027 | 0.021 | 0.127 | 0.007 | 0.044 | | | | 0.023 | | -0.001 | 0.089 | 0.059 | |
| Vocalisers weight*speech type | | *1 vs. 6 | -0.153 | 0.025 | -0.203 | -0.103 | < .001 | -0.018 | | | | 0.025 | | -0.067 | 0.030 | 0.466 | |
|  |  | *2 vs. 6 | -0.123 | 0.026 | -0.174 | -0.073 | < .001 | -0.037 | | | | 0.025 | | -0.086 | 0.011 | 0.132 | |
|  |  | *3 vs. 6 | -0.140 | 0.026 | -0.190 | -0.090 | < .001 | -0.016 | | | | 0.025 | | -0.064 | 0.032 | 0.515 | |
|  |  | *4 vs. 6 | -0.003 | 0.026 | -0.053 | 0.047 | 0.897 | -0.011 | | | | 0.025 | | -0.060 | 0.037 | 0.649 | |
|  |  | *5 vs. 6 | -0.063 | 0.026 | -0.113 | -0.012 | 0.014 | 0.001 | | | | 0.025 | | -0.048 | 0.049 | 0.972 | |
| Vocaliser’s age | |  | 0.020 | 0.002 | 0.015 | 0.024 | < .001 | 0.010 | | | | 0.002 | | 0.006 | 0.015 | < .001 | |
| Listener’s sex | |  | 0.012 | 0.028 | -0.044 | 0.067 | 0.679 | -0.060 | | | | 0.034 | | -0.126 | 0.007 | 0.078 | |
| Listener’s age | |  | -0.079 | 0.028 | -0.135 | -0.023 | 0.006 | -0.059 | | | | 0.034 | | -0.126 | 0.007 | 0.081 | |
| Random effects |  | | Variance |  | ICC |  |  | Variance | | | |  | ICC | |  | |  |
| Listener’s ID |  | | 0.267 |  | 0.310 |  |  | 0.389 | | | |  | 0.415 | |  | |  |
| Vocalizer’s ID |  | | 0.061 |  | 0.093 |  |  | 0.056 | | | |  | 0.093 | |  | |  |
| Residuals |  | | 0.595 |  |  |  |  | 0.549 | | | |  |  | |  | |  |

| Table 15. Standardized differences between perceived weight of the vocalizers across speech types in females. | | | | | | | | | | | | | | | |
| --- | --- | --- | --- | --- | --- | --- | --- | --- | --- | --- | --- | --- | --- | --- | --- |
| **Comparison** | | | | | |  | | | | | | | | | |
| **Speech type** | |  | | **Speech type** | | **Difference** | | **SE** | | **t** | | **df** | | **p_bonferroni_** | |
| single vowels |  | - |  | vowels |  | 0.020 |  | 0.026 |  | 0.780 |  | 10494.411 |  | 1.000 |  |
| single vowels |  | - |  | single word |  | 0.028 |  | 0.026 |  | 1.097 |  | 10494.411 |  | 1.000 |  |
| single vowels |  | - |  | counting |  | -0.077 |  | 0.026 |  | -3.008 |  | 10494.411 |  | 0.040 |  |
| single vowels |  | - |  | greeting |  | -0.079 |  | 0.026 |  | -3.100 |  | 10494.411 |  | 0.029 |  |
| single vowels |  | - |  | Rainbow Passage |  | -0.147 |  | 0.026 |  | -5.743 |  | 10494.411 |  | < .001 |  |
| vowels |  | - |  | single word |  | 0.008 |  | 0.026 |  | 0.316 |  | 10493.683 |  | 1.000 |  |
| vowels |  | - |  | counting |  | -0.097 |  | 0.026 |  | -3.781 |  | 10493.683 |  | 0.002 |  |
| vowels |  | - |  | greeting |  | -0.099 |  | 0.026 |  | -3.873 |  | 10493.683 |  | 0.002 |  |
| vowels |  | - |  | Rainbow Passage |  | -0.167 |  | 0.026 |  | -6.511 |  | 10493.683 |  | < .001 |  |
| single word |  | - |  | counting |  | -0.105 |  | 0.026 |  | -4.097 |  | 10493.683 |  | < .001 |  |
| single word |  | - |  | greeting |  | -0.107 |  | 0.026 |  | -4.190 |  | 10493.683 |  | < .001 |  |
| single word |  | - |  | Rainbow Passage |  | -0.175 |  | 0.026 |  | -6.827 |  | 10493.683 |  | < .001 |  |
| counting |  | - |  | greeting |  | -0.002 |  | 0.026 |  | -0.092 |  | 10493.683 |  | 1.000 |  |
| counting |  | - |  | Rainbow Passage |  | -0.070 |  | 0.026 |  | -2.730 |  | 10493.683 |  | 0.095 |  |
| greeting |  | - |  | Rainbow Passage |  | -0.068 |  | 0.026 |  | -2.638 |  | 10493.683 |  | 0.125 |  |
|  | | | | | | | | | | | | | | | |

| Table 16. Estimated marginal means of perceived weight of the vocalizers depending on speech type in females. | | | | | | | | | | | |
| --- | --- | --- | --- | --- | --- | --- | --- | --- | --- | --- | --- |
|  | | | | | | | | **95% Confidence Interval** | | | |
| **Speech type** | | **Mean** | | **SE** | | **df** | | **Lower** | | **Upper** | |
| single vowels |  | 62.318 |  | 0.378 |  | 426.908 |  | 61.575 |  | 63.061 |  |
| vowels |  | 62.134 |  | 0.378 |  | 428.100 |  | 61.390 |  | 62.877 |  |
| single word |  | 62.059 |  | 0.378 |  | 428.100 |  | 61.315 |  | 62.802 |  |
| counting |  | 63.031 |  | 0.378 |  | 428.100 |  | 62.287 |  | 63.774 |  |
| greeting |  | 63.052 |  | 0.378 |  | 428.100 |  | 62.309 |  | 63.796 |  |
| Rainbow Passage |  | 63.678 |  | 0.378 |  | 428.100 |  | 62.935 |  | 64.421 |  |
|  | | | | | | | | | | | |

| Table 17. Standardized differences between perceived weight of the vocalizers across speech types in males. | | | | | | | | | | | | | | | |
| --- | --- | --- | --- | --- | --- | --- | --- | --- | --- | --- | --- | --- | --- | --- | --- |
| **Comparison** | | | | | |  | | | | | | | | | |
| **Speech type** | |  | | **Speech type** | | **Difference** | | **SE** | | **t** | | **df** | | **p_bonferroni_** | |
| single vowels |  | - |  | vowels |  | -0.044 |  | 0.025 |  | -1.793 |  | 10301.429 |  | 1.000 |  |
| single vowels |  | - |  | single word |  | 0.077 |  | 0.025 |  | 3.099 |  | 10301.429 |  | 0.029 |  |
| single vowels |  | - |  | counting |  | -0.032 |  | 0.025 |  | -1.300 |  | 10301.429 |  | 1.000 |  |
| single vowels |  | - |  | greeting |  | -0.008 |  | 0.025 |  | -0.326 |  | 10301.429 |  | 1.000 |  |
| single vowels |  | - |  | Rainbow Passage |  | -0.054 |  | 0.025 |  | -2.177 |  | 10301.429 |  | 0.443 |  |
| vowels |  | - |  | single word |  | 0.121 |  | 0.025 |  | 4.904 |  | 10300.522 |  | < .001 |  |
| vowels |  | - |  | counting |  | 0.012 |  | 0.025 |  | 0.494 |  | 10300.522 |  | 1.000 |  |
| vowels |  | - |  | greeting |  | 0.036 |  | 0.025 |  | 1.471 |  | 10300.522 |  | 1.000 |  |
| vowels |  | - |  | Rainbow Passage |  | -0.010 |  | 0.025 |  | -0.385 |  | 10300.522 |  | 1.000 |  |
| single word |  | - |  | counting |  | -0.109 |  | 0.025 |  | -4.410 |  | 10300.522 |  | < .001 |  |
| single word |  | - |  | greeting |  | -0.085 |  | 0.025 |  | -3.433 |  | 10300.522 |  | 0.009 |  |
| single word |  | - |  | Rainbow Passage |  | -0.131 |  | 0.025 |  | -5.289 |  | 10300.522 |  | < .001 |  |
| counting |  | - |  | greeting |  | 0.024 |  | 0.025 |  | 0.977 |  | 10300.522 |  | 1.000 |  |
| counting |  | - |  | Rainbow Passage |  | -0.022 |  | 0.025 |  | -0.879 |  | 10300.522 |  | 1.000 |  |
| greeting |  | - |  | Rainbow Passage |  | -0.046 |  | 0.025 |  | -1.856 |  | 10300.522 |  | 0.952 |  |
|  | | | | | | | | | | | | | | | |

| Table 18. Estimated marginal means of perceived weight of the vocalizers depending on speech type in males. | | | | | | | | | | | |
| --- | --- | --- | --- | --- | --- | --- | --- | --- | --- | --- | --- |
|  | | | | | | | | **95% Confidence Interval** | | | |
| **Speech type** | | **Mean** | | **SE** | | **df** | | **Lower** | | **Upper** | |
| single vowels |  | 76.292 |  | 0.495 |  | 518.454 |  | 75.321 |  | 77.264 |  |
| vowels |  | 76.794 |  | 0.494 |  | 516.812 |  | 75.823 |  | 77.764 |  |
| single word |  | 75.426 |  | 0.494 |  | 516.812 |  | 74.455 |  | 76.397 |  |
| counting |  | 76.656 |  | 0.494 |  | 516.812 |  | 75.685 |  | 77.627 |  |
| greeting |  | 76.383 |  | 0.494 |  | 516.812 |  | 75.412 |  | 77.354 |  |
| Rainbow Passage |  | 76.901 |  | 0.494 |  | 516.812 |  | 75.930 |  | 77.872 |  |
| Note. Estimated means are estimated keeping constant other effects in the model to the mean | | | | | | | | | | | |
|  | | | | | | | | | | | |

Table 19. Significance of differences between the individual correlation coefficients. The values were extracted using an online app: <http://quantpsy.org/corrtest/corrtest2.htm>. Significant differences are bolded.

|  |  | Z - value  Female vocalisers | P -value  Female  vocalisers | Z - value  Male vocalisers | P -value  Male  vocalisers |
| --- | --- | --- | --- | --- | --- |
| **height** |  |  |  |  |  |
| single vowels | vowels | -0.324 | 0.746 | -0.672 | 0.502 |
|  | words | 0.641 | 0.521 | 0.419 | 0.675 |
|  | counting | 0.398 | 0.690 | -0.23 | 0.818 |
|  | greeting | -1.016 | 0.310 | -0.346 | 0.730 |
|  | Rainbow Passage | -0.202 | 0.840 | 0.231 | 0.817 |
| vowels | words | 0.83 | 0.407 | 1.079 | 0.281 |
|  | counting | 0.714 | 0.475 | 0.523 | 0.601 |
|  | greeting | 0.679 | 0.497 | 0.377 | 0.706 |
|  | Rainbow Passage | 0.117 | 0.907 | 0.959 | 0.338 |
| words | counting | -0.095 | 0.924 | -0.687 | 0.492 |
|  | greeting | -0.09 | 0.929 | -0.759 | 0.448 |
|  | Rainbow Passage | -0.753 | 0.452 | -0.211 | 0.833 |
| counting | greeting | 0 | 1 | -0.146 | 0.884 |
|  | Rainbow Passage | -0.777 | 0.437 | 0.525 | 0.600 |
| greeting | Rainbow Passage | -0.707 | 0.480 | 0.758 | 0.448 |
| **age** |  |  |  |  |  |
| single vowels | vowels | -1.333 | 0.182 | **-2.999** | **0.003** |
|  | words | 0.52 | 0.603 | -0.5 | 0.617 |
|  | counting | **-3.399** | **0.001** | **-3.245** | **0.001** |
|  | greeting | **-3.8** | **0.0001** | **-3.214** | **0.001** |
|  | Rainbow Passage | **-5.458** | **0** | **-4.166** | **0.000031** |
| vowels | words | 1.727 | 0.084 | 1.683 | 0.092 |
|  | counting | **-3.137** | **0.002** | -1.645 | 0.100 |
|  | greeting | **-3.104** | **0.002** | -1.459 | 0.144531 |
|  | Rainbow Passage | **-2.682** | **0** | **-3.742** | **0.007321** |
| words | counting | **-3.78** | **0.0002** | **-3.742** | **0.001825** |
|  | greeting | **-4.09** | **0.00004** | **-3.117** | **0.001825** |
|  | Rainbow Passage | **-5.789** | **0** | **-4.425** | **0.00001** |
| counting | Greeting | -0.558 | 0.577 | 0.306 | 0.760 |
|  | Rainbow Passage | **-2.838** | **0.005** | -1.375 | 0.169 |
| greeting | Rainbow Passage | **-1.978** | **0.048** | -1.364 | 0.172 |
| **weight** |  |  |  |  |  |
| single vowels | vowels | -1.016 | 0.310 | 0.729 | 0.466 |
|  | words | -0.351 | 0.725 | 0 | 1 |
|  | counting | **-2.94** | **0.003** | -0.124 | 0.901 |
|  | greeting | -1.718 | 0.086 | -0.46 | 0.646 |
|  | Rainbow Passage | **-2.853** | **0.004** | -0.362 | 0.717 |
| vowels | words | 0.561 | 0.575 | -0.719 | 0.472 |
|  | counting | **-2.666** | **0.008** | -0.863 | 0.388 |
|  | greeting | -0.971 | 0.332 | -1.158 | 0.247 |
|  | Rainbow Passage | **-2.491** | **0.013** | -1.113 | 0.266 |
| words | counting | **-3.318** | **0.001** | -0.138 | 0.890 |
|  | Greeting | -1.538 | 0.124 | -0.537 | 0.591 |
|  | Rainbow Passage | -2.631 | 0.009 | -0.384 | 0.701 |
| counting | greeting | **1.986** | **0.047** | -0.417 | 0.677 |
|  | Rainbow Passage | 0.226 | 0.821 | -0.337 | 0.736 |
| greeting | Rainbow Passage | -1.725 | 0.084 | 0.145 | 0.885 |
